# Dopamine and Serotonin Are Both Required for Mate-Copying in *Drosophila melanogaster*

**DOI:** 10.3389/fnbeh.2018.00334

**Published:** 2019-01-09

**Authors:** Magdalena Monier, Sabine Nöbel, Etienne Danchin, Guillaume Isabel

**Affiliations:** ^1^Laboratoire Évolution & Diversité Biologique, UMR5174, CNRS, IRD, Université Toulouse III – Paul Sabatier, Toulouse, France; ^2^Institute for Advanced Study in Toulouse, Toulouse, France; ^3^Centre de Recherches sur la Cognition Animale (CRCA), Centre de Biologie Intégrative (CBI), Université de Toulouse, CNRS, UPS, Toulouse, France

**Keywords:** fruit fly, mate choice, social learning, social memory, 3-iodotyrosine (3-IY), DL-para-chlorophenylalanine (PCPA), L-3, 4-dihydroxyphenylalanine (L-DOPA), 5-L-hydroxytryptophan (5-HTP)

## Abstract

Mate-copying is a form of social learning in which the mate-choice decision of an individual (often a female) is influenced by the mate-choice of conspecifics. *Drosophila melanogaster* females are known to perform such social learning, and in particular, to mate-copy after a single observation of one conspecific female mating with a male of one phenotype, while the other male phenotype is rejected. Here, we show that this form of social learning is dependent on serotonin and dopamine. Using a pharmacological approach, we reduced dopamine or serotonin synthesis in adult virgin females with 3-iodotyrosine (3-IY) and DL-para-chlorophenylalanine (PCPA), respectively, and then tested their mate-copying performance. We found that, while control females without drug treatment copied the choice of the demonstrator, drug-treated females with reduced dopamine or serotonin chose randomly. To ensure the specificity of the drugs, the direct precursors of the neurotransmitters, either the dopamine precursor L-3,4-dihydroxyphenylalanine (L-DOPA) or the serotonin precursor 5-L-hydroxytryptophan (5-HTP) were given together with the drug, (respectively 3-IY and PCPA) resulting in a full rescue of the mate-copying defects. This indicates that dopamine and serotonin are both required for mate-copying. These results give a first insight into the mechanistic pathway underlying this form of social learning in *D. melanogaster*.

## Introduction

Many animal species from a vast array of taxa can learn from others (i.e., social learning), particularly in the context of mate-choice (Avital and Jablonka, 2000; Danchin et al., 2004; Galef and Laland, 2005). Such observational learning can lead to mate-copying (Pruett-Jones, 1992), when females mate preferentially with a male showing similar characteristics as the male they saw being chosen by another female (trait-based copying, Bowers et al., 2012).

In *Drosophila melanogaster*, females are able to perform mate-copying (Mery et al., 2009) after watching only a single live demonstration of one female copulating with a male of a given phenotype and one male of another phenotype being rejected (Dagaeff et al., 2016; Danchin et al., 2018; Nöbel et al., 2018).

Despite some promising studies, research about the mechanisms of social learning in general and observational social learning in particular are still at the beginning (Burke et al., 2010; Debiec and Olsson, 2017; Kavaliers et al., 2017; Allsop et al., 2018). While social learning mechanisms are poorly known in any organism, *D. melanogaster* with its mini yet highly structured brain (100,000 cells) is one of the most favorable model species to dissect the neuronal processes of learning. Mostly, studies focused on simple kinds of learning tasks, where flies can learn from their own experience (non-social learning task), that are easier to standardize and historically well studied, like olfactory or visual associative learning (Quinn et al., 1974; Vogt et al., 2014, 2016; Cognigni et al., 2018). Thus, while the mechanisms of non-social learning in *Drosophila* are now well-described, the neurotransmitters and neural structures involved in observational social learning in *Drosophila* are unknown.

The formation of non-social associative memory requires dopamine in *D. melanogaster*: during the olfactory or visual learning process, it mediates aversive or appetitive unconditional stimuli (Riemensperger et al., 2005, 2011; Aso et al., 2012; Burke et al., 2012; Liu et al., 2012; Vogt et al., 2014), while serotonin is required for aversive place memory (Sitaraman et al., 2008), and for olfactory learning and memory (Johnson et al., 2011; Lee et al., 2011). Based on the fact that visual and olfactory learning share common neurotransmitters and neural structures (Vogt et al., 2014), we hypothesized that our model of observational social learning, mate-copying, involves the same two neurotransmitters. To address this, we used a pharmacological approach to reduce dopamine or serotonin synthesis with specific inhibitors of the limiting-step-enzyme of the synthetic pathway: 3-iodotyrosine (3-IY) inhibits tyrosine hydroxylase that catalyzes L-DOPA formation from tyrosine, and DL-para-chlorophenylalanine (PCPA) inhibits tryptophan hydroxylase that catalyzes 5-HTP formation from tryptophan, respectively. Young sexually mature virgin females were fed one of these drugs and their mate-copy ability was tested after a single demonstration. To ensure specificity of the drugs, we also had two rescue treatments in which the female received the drug (3-IY or PCPA) together with the immediate precursor of the neurotransmitter (L-DOPA or 5-HTP, respectively), so that the level of dopamine or serotonin was less reduced than with 3-IY or PCPA alone.

## Materials and Methods

### Fly Maintenance

Wild-type Canton-S flies were raised in 30 ml food vials containing standard corn flour-yeast-agar medium. The room was maintained at 25 ± 0.8°C, 60 ± 3% humidity, with a 12 h:12 h light:dark cycle. Virgin flies were collected daily for the experiments and sexed without anesthesia, by gentle aspiration using a glass pipette, tubing and gauze. Flies were then kept in single-sex groups in food vials until the experiment started. As *D. melanogaster* females are reluctant to re-mate (Chapman et al., 2003), each female was used only once as demonstrator or observer.

### Drug Treatment

The solutions were freshly prepared every week in vehicle (sucrose 5% in mineral water Vittel^®^) and 200 ml were poured on a Kimwipe paper (1.5 cm × 3.5 cm) deposited in a 15 ml Falcon tube. Nine 1-day-old virgin females were introduced in the tube for the length of the treatment, at 18°C, 12 h:12 h light:dark cycle. To explore dopamine effect, flies were fed with 3-IY (10 mg/ml, Sigma I8250) and/or L-DOPA (1 mg/ml, Sigma D9628) for 36–40 h (Bainton et al., 2000; Seugnet et al., 2008). To explore serotonin effect, flies were fed with PCPA (10 mg/ml, Sigma C6506) and/or 5-HTP (16 mg/ml, Sigma H9772) for 3 days, with papers being changed once during the 3 days period (Dierick and Greenspan, 2007; Plaçais et al., 2012). We used a high 5-HTP concentration (30% more than in Dierick and Greenspan, 2007) to ensure rescued serotonin levels in PCPA-treated flies in our conditions. This concentration did not affect mate-copying in flies fed with 5-HTP (Figure 2). The treatment “vehicle” consisted of vehicle solution given during 36–40 h or 3 days.

### Mate-Copying Experiment

Flies were tested 3–4 days after eclosion. Experiments were conducted in the same conditions as fly maintenance. We used the same tubes set-up (double plastic tube (0.8 × 3 cm each) separated by a thin glass partition) and the speed-learning protocol as described in Dagaeff et al. (2016) except that mate-choice tests were run either immediately to test learning, or 3 h 20 ± 15 min after the demonstration, a time when associative memory in drosophila is composed of consolidated and labile memories (Folkers et al., 1993), two memories with independent pathways (Isabel et al., 2004; Scheunemann et al., 2012) so that we could detect a learning and/or memory defect. Artificial male phenotypes were obtained by randomly dusting them in green or pink (neutral trait) using colored powders (green: Shannon Luminous Materials, Inc., #B-731; red: BioQuip Products, Inc., #1162R). Demonstrations in tube set-ups showed a demonstrator female choosing between the two male phenotypes while the treated female could observe through a transparent partition. After the end of the copulation of demonstrator flies, each observer female was either directly tested or placed individually in a food vial until the test. The mate-choice test then involved two new virgin green and pink males placed in a tube with the observer female. Time when courtship began (first wing vibration) and color of the male, as well as time when copulation started and color of the chosen male were recorded.

### Mate-Copying Index

Observer females that chose the same male color as the demonstrator for copulation (copied) were given a mate-copying score of 1, and females that chose the opposite color were given a score of 0. A mate-copying index (MCI) was calculated as the mean mate-copying score per treatment. Samples in which only one male courted the female before she initiated mating were not used for the analysis of the mate-copying performance because only when both males showed their interest the female was unambiguously in a position of choice. Samples in which no copulation occurred after 30 min were excluded from the analyses.

### Ethics Statement

Behavioral observations of *D. melanogaster* required no ethical approval and complied with French laws regarding animal welfare. We kept the number of flies used in this study as small as possible. We handled flies by gentle aspiration without anesthesia to minimize damage and discomfort. After the experiments, individuals were euthanized in a freezer.

### Analyses

Mate-copying scores were analyzed with the R software 3.4.0 (R Core Team, 2017). For each treatment, the difference from random choice was tested with a binomial test. For global comparisons, mate-copying scores were analyzed in a generalized linear mixed model (GLMM) with binary logistic regression (package lme4, Bates et al., 2014). A random block effect introduced into the models accounted for the non-independence of observer flies from the same block of 6 demonstrations and tests. The significance of fixed effects was tested using Wald chi-square tests implemented in the ANOVA function of the car package (Fox and Weisberg, 2011). Starting models included treatment, air pressure at the time of the test, and its variation within the 6 preceding hours, and interactions between these effects. We used a backward selection approach using *P*-values, removing the highest order interaction as soon as it was non-significant. The final model was always chosen as the one with the lowest Akaike Information Criteria (AIC, Akaike, 1969). Two-by-two comparisons between treatments were done using *post hoc* *X*^2^ tests.

## Results

### PCPA and 3-IY Impair Learning or Memory in Mate-Copying

**FIGURE 1:**
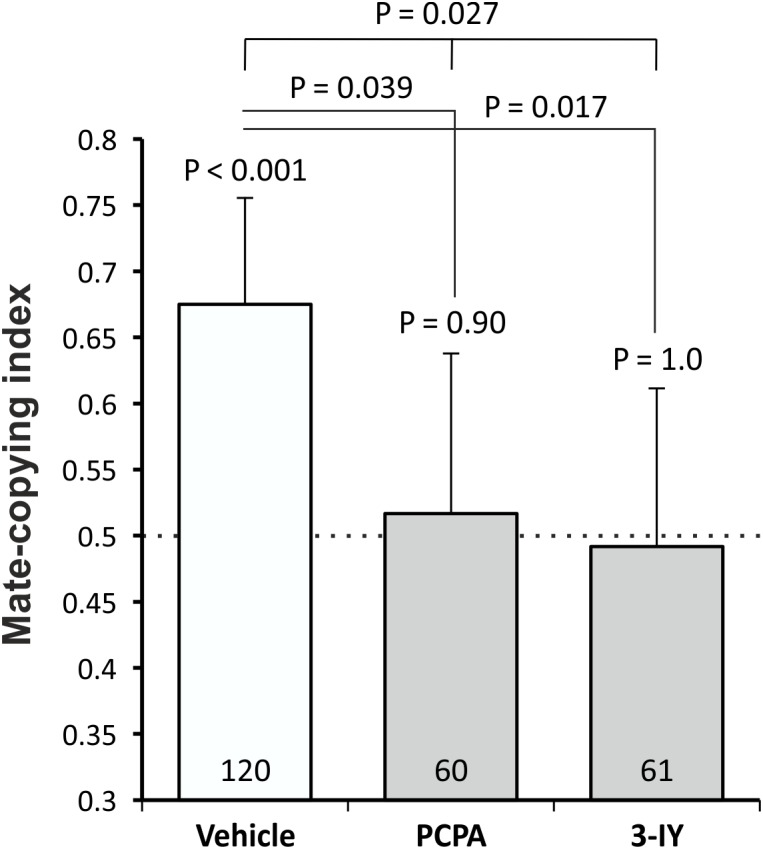
Mate-copying index measured 3 h after the demonstration. Numbers inside the bars represent the sample size. Dashed line indicates random choice. Error bars: Agresti-Coull intervals. Just above bars: binomial tests. Two-by-two comparisons: *post hoc* chi-square tests. Test between all groups: GLMM.

We first tested whether females’ mate-copying performance was affected after a PCPA or a 3-IY treatment. We analyzed the mate-copying scores (Figure 1) and found that females fed with the vehicle mate-copied, while females lacking serotonin or dopamine did not. We then compared the three groups and found a significant difference (GLMM, *N* = 241, *X*^2^ = 7.26, *P* = 0.027), which we also found when comparing PCPA- or 3-IY-treated flies to the vehicle (Figure 1 and Supplementary Table S1). Thus, PCPA and 3-IY impaired mate-copying in these conditions. We also measured courtship duration in each group and found no statistical difference between them (Supplementary Figure S1).

### Dopamine and Serotonin Are Both Required for Learning in a Mate-Copying Context

**FIGURE 2:**
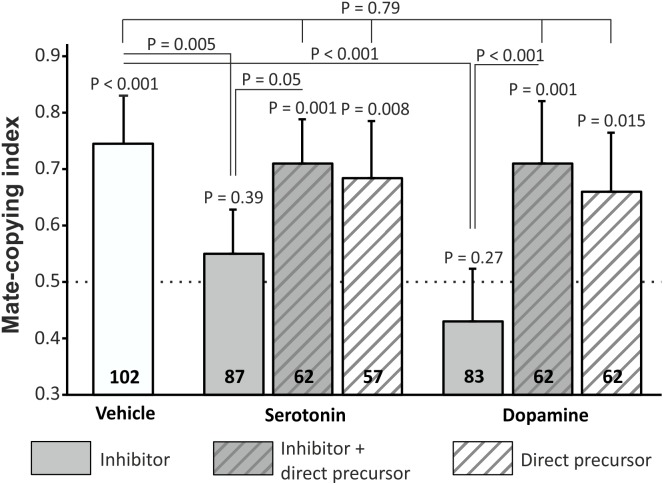
Mate-copying index measured immediately after the demonstration. Flies received the following treatment (from left to right): vehicle, PCPA, PCPA + 5-HTP, 5-HTP, 3-IY, 3-IY + L-DOPA, L-DOPA. Numbers inside the bars represent the sample size. Dashed line indicates random choice. Error bars: Agresti-Coull intervals. Just above bars: binomial tests. Two-by-two comparisons: *post hoc* chi-square tests. Test between all groups that copied: GLMM.

We then tested female mate-copying immediately after the demonstration, in order to study learning capacities only, and not memory retention. To ensure that the mate-copying defects observed in Figure 1 depend on lacking dopamine or serotonin, and not to a side-effect of the drug, we added four more treatments: PCPA with 5-HTP, 5-HTP, 3-IY with L-DOPA and L-DOPA. We measured mate-copying scores in all groups (Figure 2) and found that all groups copied except PCPA and 3-IY treated females. We compared mate-copying scores in the five groups that copied and found no statistical difference (GLMM, *N* = 345, *X*^2^ = 1.72, *P* = 0.79), indicating that 5-HTP and L-DOPA given alone did not alter mate-copying ability, and could rescue mate-copying in females treated with the inhibitor. Additionally, we found a significant difference between inhibitor-treated flies and flies fed with the vehicle or rescued flies (Figure 2 and Supplementary Table S2). Thus, flies lacking dopamine or serotonin are not able to learn a mate preference from the demonstration.

## Discussion

We found that dopamine and serotonin are both required in learning during mate-copying. Observer females lacking these neurotransmitters were unable to learn the successful male phenotype in the demonstration while control females receiving the vehicle solution, females treated with 5-HTP or L-DOPA, and females treated with the precursor together with the inhibitor copied the choice of the demonstrator immediately after the demonstration. This is in accordance with other studies showing that dopamine and serotonin are required for learning. In an olfactory learning task, dopamine is required to mediate the unconditional stimulus after a single training phase (Riemensperger et al., 2011; Aso et al., 2012; Burke et al., 2012; Liu et al., 2012; Lin et al., 2014; Vogt et al., 2014). Alterations in behavioral tracking were reported in flies lacking dopamine (Andretic et al., 2005), but dopamine-deficient flies were shown to have no alteration in visual perception and display a normal electroretinogram (Riemensperger et al., 2011). Thus, the defects we observed are not due to deficient vision, although we cannot exclude attention deficiency in dopamine-depleted flies. Serotonin is necessary to form place memory (Sitaraman et al., 2008) and associative olfactory learning memory (Johnson et al., 2011; Lee et al., 2011). Mate-copying can be compared to associative learning with pairing between a conditional and an unconditional stimulus (Avarguès-Weber et al., 2015): the conditional stimulus would be the color of the male copulating with the demonstrator female while the unconditional reinforcing stimulus could be the observation of the copulation. Under these circumstances, dopamine would mediate the reinforcing stimulus. Our results provide one more indication that the pathways underlying memory formation are comparable for visual social information and for olfactory information, and it was shown that both share mushroom body circuits for memory consolidation (Vogt et al., 2014). Mate-copying was also described in many vertebrates (Dugatkin and Godin, 1993; White and Galef, 1999; Waynforth, 2007; Galef et al., 2008), so the mechanistic results discovered in *Drosophila* could be a starting point for such studies in vertebrates, as many vertebrate pathways and genes have homologs in *Drosophila*.

We showed that dopamine and serotonin are both required in mate-copying. This result paves the way for further studies of the neural pathways underlying social observational learning in *D. melanogaster*. The next step is now to dig into the role of each of these neurotransmitters, by assessing the neural structures and the receptors involved in this social learning task.

## Data Availability

The raw data supporting the conclusions of this manuscript will be made available by the authors, without undue reservation, to any qualified researcher.

## Author Contributions

MM carried out the experiments, performed the analyses, and drafted the manuscript. SN contributed in the writing of the manuscript. ED and GI designed the experiments and jointly supervised all steps in the process. All authors gave final approval for publication.

## Conflict of Interest Statement

The authors declare that the research was conducted in the absence of any commercial or financial relationships that could be construed as a potential conflict of interest.

## References

[B1] Akaike H. (1969). Fitting autoregressive models for prediction. *Ann. Inst. Stat. Math.* 21 243–247. 10.1007/BF02532251

[B2] Allsop S. A. Wichmann R. Mills F. Burgos-Robles A. Chang C.-J. Felix-Ortiz A. C. (2018). Corticoamygdala transfer of socially derived information gates observational learning. *Cell* 173 1329.e18–1342.e18. 10.1016/j.cell.2018.04.004 29731170 PMC6345560

[B3] Andretic R. van Swinderen B. Greenspan R. J. (2005). Dopaminergic modulation of arousal in *Drosophila*. *Curr. Biol.* 15 1165–1175. 10.1016/j.cub.2005.05.025 16005288

[B4] Aso Y. Herb A. Ogueta M. Siwanowicz I. Templier T. Friedrich A. B. (2012). Three dopamine pathways induce aversive odor memories with different stability. *PLoS Genet.* 8:e1002768. 10.1371/journal.pgen.1002768 22807684 PMC3395599

[B5] Avarguès-Weber A. Lihoreau M. Isabel G. Giurfa M. (2015). Information transfer beyond the waggle dance: observational learning in bees and flies. *Front. Ecol. Evol.* 3:24 10.3389/fevo.2015.00024

[B6] Avital E. Jablonka E. (2000). *Animal Traditions: Behavioural Inheritance in Evolution*. Cambridge: Cambridge University Press 10.1017/CBO9780511542251 15307257

[B7] Bainton R. J. Tsai L. T.-Y. Singh C. M. Moore M. S. Neckameyer W. S. Heberlein U. (2000). Dopamine modulates acute responses to cocaine, nicotine and ethanol in *Drosophila*. *Curr. Biol.* 10 187–194. 10.1016/S0960-9822(00)00336-5 10704411

[B8] Bates D. Mächler M. Bolker B. Walker S. (2014). Fitting Linear Mixed-Effects Models using lme4. *arXiv* [preprint]. arXiv:1406.5823

[B9] Bowers R. I. Place S. S. Todd P. M. Penke L. Asendorpf J. B. (2012). Generalization in mate-choice copying in humans. *Behav. Ecol.* 23 112–124. 10.1093/beheco/arr164

[B10] Burke C. J. Huetteroth W. Owald D. Perisse E. Krashes M. J. Das G. (2012). Layered reward signalling through octopamine and dopamine in *Drosophila*. *Nature* 492 433–437. 10.1038/nature11614 23103875 PMC3528794

[B11] Burke C. J. Tobler P. N. Baddeley M. Schultz W. (2010). Neural mechanisms of observational learning. *Proc. Natl. Acad. Sci. U.S.A.* 107 14431–14436. 10.1073/pnas.1003111107 20660717 PMC2922583

[B12] Chapman T. Bangham J. Vinti G. Seifried B. Lung O. Wolfner M. F. (2003). The sex peptide of *Drosophila melanogaster*: female post-mating responses analyzed by using RNA interference. *Proc. Natl. Acad. Sci. U.S.A.* 100 9923–9928. 10.1073/pnas.1631635100 12893873 PMC187888

[B13] Cognigni P. Felsenberg J. Waddell S. (2018). Do the right thing: neural network mechanisms of memory formation, expression and update in *Drosophila*. *Curr. Opin. Neurobiol.* 49 51–58. 10.1016/j.conb.2017.12.002 29258011 PMC5981003

[B14] Dagaeff A.-C. Pocheville A. Nöbel S. Loyau A. Isabel G. Danchin E. (2016). *Drosophila* mate copying correlates with atmospheric pressure in a speed learning situation. *Anim. Behav.* 121 163–174. 10.1016/j.anbehav.2016.08.022

[B15] Danchin E. Nöbel S. Pocheville A. Dagaeff A.-C. Demay L. Alphand M. (2018). Cultural flies: conformist social learning in fruit flies predicts long-lasting mate-choice traditions. *Science* 362 1025–1030. 10.1126/science.aat1590 30498121

[B16] Danchin É Giraldeau L.-A. Valone T. J. Wagner R. H. (2004). Public information: from nosy neighbors to cultural evolution. *Science* 305 487–491. 10.1126/science.1098254 15273386

[B17] Debiec J. Olsson A. (2017). Social fear learning: from animal models to human function. *Trends Cogn. Sci.* 21 546–555. 10.1016/j.tics.2017.04.010 28545935 PMC5507357

[B18] Dierick H. A. Greenspan R. J. (2007). Serotonin and neuropeptide F have opposite modulatory effects on fly aggression. *Nat. Genet.* 39 678–682. 10.1038/ng2029 17450142

[B19] Dugatkin L. A. Godin J.-G. J. (1993). Female mate copying in the guppy (*Poecilia reticulata*): age-dependent effects. *Behav. Ecol.* 4 289–292. 10.1093/beheco/4.4.289

[B20] Folkers E. Drain P. Quinn W. G. (1993). Radish, a *Drosophila* mutant deficient in consolidated memory. *Proc. Natl. Acad. Sci. U.S.A.* 90 8123–8127. 10.1073/pnas.90.17.8123 8367473 PMC47300

[B21] Fox J. Weisberg S. (2011). *An {R} Companion to Applied Regression.* 2nd Edn. Thousand Oaks, CA: Sage Publishing.

[B22] Galef B. G. Laland K. N. (2005). Social learning in animals: empirical studies and theoretical models. *Bioscience* 55 489–499. 10.1641/0006-3568(2005)055[0489:SLIAES]2.0.CO;2

[B23] Galef B. G. Lim T. C. W. Gilbert G. S. (2008). Evidence of mate choice copying in Norway rats, *Rattus norvegicus*. *Anim. Behav.* 75 1117–1123. 10.1016/j.anbehav.2007.08.026

[B24] Isabel G. Pascual A. Preat T. (2004). Exclusive consolidated memory phases in *Drosophila*. *Science* 304 1024–1027. 10.1126/science.1094932 15143285

[B25] Johnson O. Becnel J. Nichols C. D. (2011). Serotonin receptor activity is necessary for olfactory learning and memory in *Drosophila melanogaster*. *Neuroscience* 192 372–381. 10.1016/j.neuroscience.2011.06.058 21749913 PMC3166404

[B26] Kavaliers M. Matta R. Choleris E. (2017). Mate-choice copying, social information processing, and the roles of oxytocin. *Neurosci. Biobehav. Rev.* 72 232–242. 10.1016/j.neubiorev.2016.12.003 27923732

[B27] Lee P.-T. Lin H.-W. Chang Y.-H. Fu T.-F. Dubnau J. Hirsh J. (2011). Serotonin–mushroom body circuit modulating the formation of anesthesia-resistant memory in *Drosophila*. *Proc. Natl. Acad. Sci. U.S.A.* 108 13794–13799. 10.1073/pnas.1019483108 21808003 PMC3158232

[B28] Lin S. Owald D. Chandra V. Talbot C. Huetteroth W. Waddell S. (2014). Neural correlates of water reward in thirsty *Drosophila*. *Nat. Neurosci.* 17 1536–1542. 10.1038/nn.3827 25262493 PMC4213141

[B29] Liu C. Plaçais P.-Y. Yamagata N. Pfeiffer B. D. Aso Y. Friedrich A. B. (2012). A subset of dopamine neurons signals reward for odour memory in *Drosophila*. *Nature* 488 512–516. 10.1038/nature11304 22810589

[B30] Mery F. Varela S. A. M. Danchin É Blanchet S. Parejo D. Coolen I. (2009). Public versus personal information for mate copying in an invertebrate. *Curr. Biol.* 19 730–734. 10.1016/j.cub.2009.02.064 19361993

[B31] Nöbel S. Danchin E. Isabel G. (2018). Mate-copying for a costly variant in *Drosophila melanogaster* females. *Behav. Ecol.* 29 1150–1156. 10.1093/beheco/ary095

[B32] Plaçais P.-Y. Trannoy S. Isabel G. Aso Y. Siwanowicz I. Belliart-Guérin G. (2012). Slow oscillations in two pairs of dopaminergic neurons gate long-term memory formation in *Drosophila*. *Nat. Neurosci.* 15 592–599. 10.1038/nn.3055 22366756

[B33] Pruett-Jones S. (1992). Independent versus nonindependent mate choice: do females copy each other? *Am. Nat.* 140 1000–1009. 10.1086/285452 19426031

[B34] Quinn W. G. Harris W. Benzer S. (1974). Conditioned Behavior in *Drosophila melanogaster*. *Proc. Natl. Acad. Sci. U.S.A.* 71 708–712. 10.1073/pnas.71.3.708 4207071 PMC388082

[B35] R Core Team (2017). *R: A Language and Environment for Statistical Computing*. Vienna: R Foundation for Statistical Computing.

[B36] Riemensperger T. Isabel G. Coulom H. Neuser K. Seugnet L. Kume K. (2011). Behavioral consequences of dopamine deficiency in the *Drosophila* central nervous system. *Proc. Natl. Acad. Sci. U.S.A.* 108 834–839. 10.1073/pnas.1010930108 21187381 PMC3021077

[B37] Riemensperger T. Völler T. Stock P. Buchner E. Fiala A. (2005). Punishment prediction by dopaminergic neurons in *Drosophila*. *Curr. Biol.* 15 1953–1960. 10.1016/j.cub.2005.09.042 16271874

[B38] Scheunemann L. Jost E. Richlitzki A. Day J. P. Sebastian S. Thum A. S. (2012). Consolidated and labile odor memory are separately encoded within the *Drosophila* brain. *J. Neurosci.* 32 17163–17171. 10.1523/jneurosci.3286-12.2012 23197709 PMC6621839

[B39] Seugnet L. Suzuki Y. Vine L. Gottschalk L. Shaw P. J. (2008). D1 Receptor activation in the mushroom bodies rescues sleep-loss-induced learning impairments in *Drosophila*. *Curr. Biol.* 18 1110–1117. 10.1016/j.cub.2008.07.028 18674913 PMC2603029

[B40] Sitaraman D. Zars M. LaFerriere H. Chen Y.-C. Sable-Smith A. Kitamoto T. (2008). Serotonin is necessary for place memory in *Drosophila*. *Proc. Natl. Acad. Sci. U.S.A.* 105 5579–5584. 10.1073/pnas.0710168105 18385379 PMC2291120

[B41] Vogt K. Aso Y. Hige T. Knapek S. Ichinose T. Friedrich A. B. (2016). Direct neural pathways convey distinct visual information to *Drosophila* mushroom bodies. *eLife* 5:e14009. 10.7554/eLife.14009 27083044 PMC4884080

[B42] Vogt K. Schnaitmann C. Dylla K. V. Knapek S. Aso Y. Rubin G. M. (2014). Shared mushroom body circuits underlie visual and olfactory memories in *Drosophila*. *eLife* 3:e02395. 10.7554/eLife.02395 25139953 PMC4135349

[B43] Waynforth D. (2007). Mate choice copying in humans. *Hum. Nat.* 18 264–271. 10.1007/s12110-007-9004-2 26181063

[B44] White D. J. Galef B. G. Jr. (1999). Mate choice copying and conspecific cueing in Japanese quail, *Coturnix coturnix* japonica. *Anim. Behav.* 57 465–473. 10.1006/anbe.1998.1015 10049487

