# Supplementary material for: Dopamine and Serotonin Are Both Required for Mate-Copying in Drosophila melanogaster
Source: Front Behav Neurosci. 2019 Jan 9;12:334. doi: 10.3389/fnbeh.2018.00334 (PMC6333735; doi:10.3389/fnbeh.2018.00334)
Supplement: Supplementary file 1 [file Data_Sheet_1.docx]

Supplementary Material

Dopamine and serotonin are both required for mate-copying in *Drosophila melanogaster*

Magdalena Monier^*^, Sabine Nöbel, Etienne Danchin^†^ & Guillaume Isabel^†^

^†^Co-senior authors.

*** Correspondence:**Magdalena Monier
magdalena.monier@univ-tlse3.fr

**Supplementary table 1: post-hoc X² tests comparing groups of flies from figure 1**

| *Groups compared* | *N* | *X²* | *P-value* |
| --- | --- | --- | --- |
| PCPA to vehicle | 180 | 4.27 | 0.039 |
| 3-IY to vehicle | 181 | 5.72 | 0.017 |

**
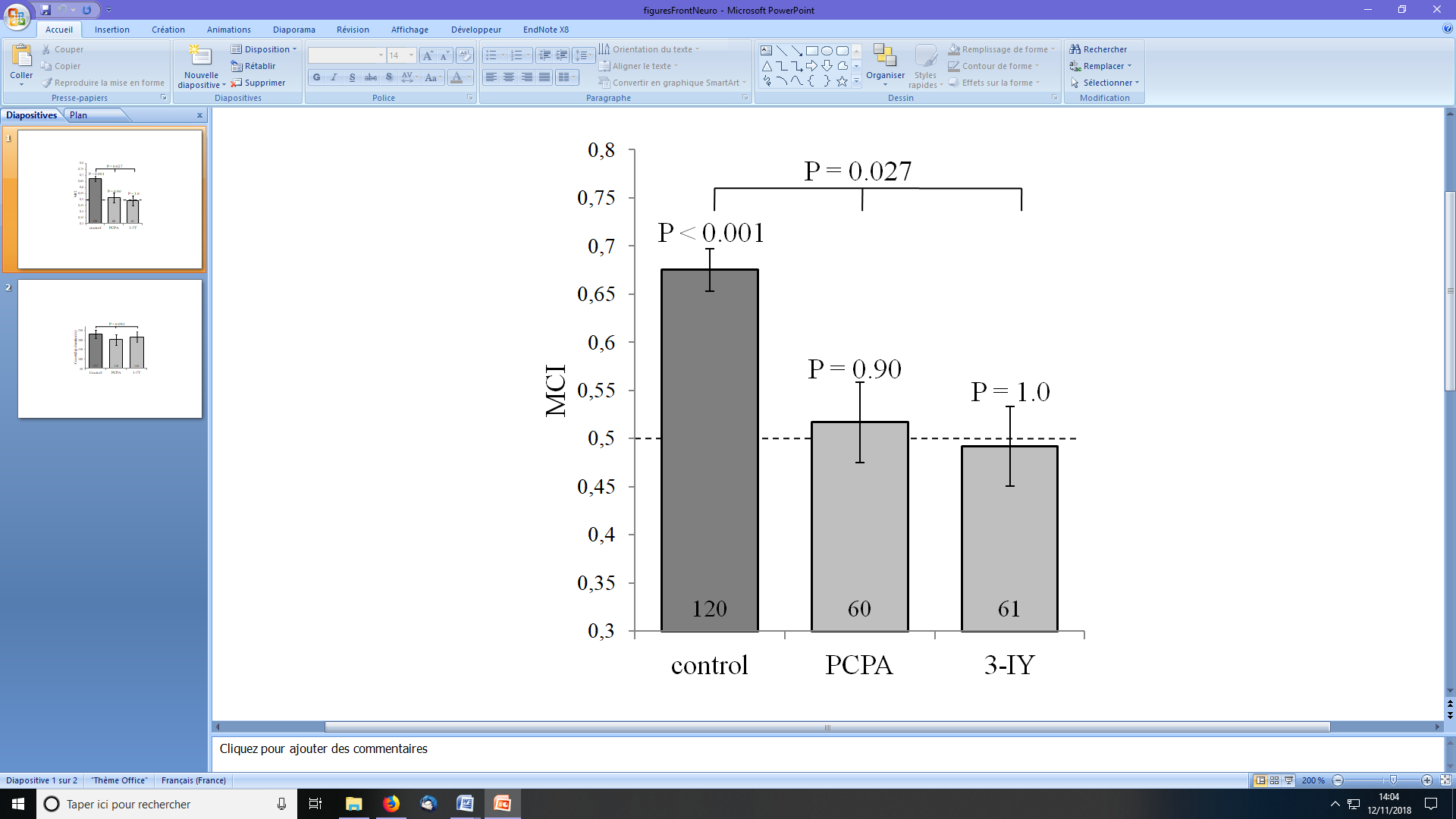
**

**Supplementary figure 1: Mean courtship duration for each treatment.** Log-transformed courtship duration was analyzed in a linear mixed model (LMM) with logistic regression. All trials with detailed times of courtship and copulation initiation were analyzed. Log-transformation (natural log) was used to achieve a Gaussian distribution of that variable. The starting model included treatment and log-transformed time when first courtship began. The selected model included this last parameter alone. Treatment effect was found non-significant (LMM, N = 476, X² = 4.73, P = 0.094), while time when first courtship began had a significant effect (P < 0.001, the later the courtship began the shorter it was).

**Supplementary table 2: post-hoc X² tests comparing groups of flies from figure 2**

| *Groups compared* | *N* | *X²* | *P-value* |
| --- | --- | --- | --- |
| PCPA to vehicle | 189 | 7.78 | 0.005 |
| PCPA to PCPA + 5-HTP | 149 | 3.82 | 0.05 |
| 3-IY to vehicle | 185 | 18.6 | <0.001 |
| 3-IY to 3-IY + L-DOPA | 145 | 10.9 | <0.001 |
